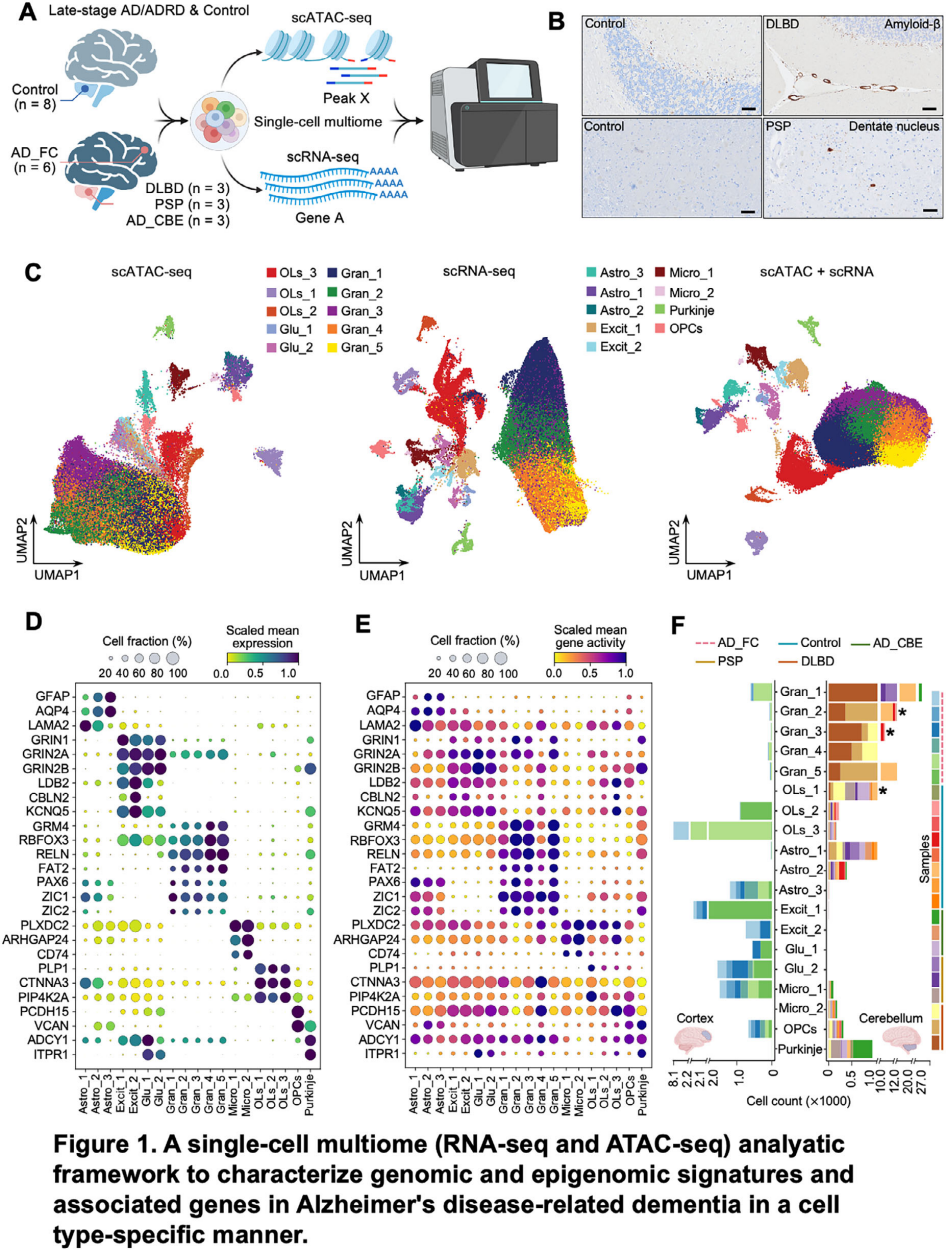# Genomic and epigenomic insights into purkinje and granule cells in Alzheimer's disease‐related dementia using single‐nucleus multiome analysis

**DOI:** 10.1002/alz70855_105855

**Published:** 2025-12-24

**Authors:** Feixiong Cheng, Yayan Feng, Margaret E Flanagan, Borna Bonakdarpour, Jeffrey L. Cummings

**Affiliations:** ^1^ Cleveland Clinic Genome Center, Cleveland, OH, USA; ^2^ Cleveland Clinic, Cleveland, OH, USA; ^3^ Northwestern ADC Neuropathology Core, Northwestern University Feinberg School of Medicine, Chicago, IL, USA; ^4^ University of Texas Health Science Center at San Antonio, San Antonio, TX, USA; ^5^ Northwestern Music and Medicine Program, Chicago, IL, USA; ^6^ Mesulam Center for Cognitive Neurology and Alzheimer's Disease, Chicago, IL, USA; ^7^ Northwestern University Feinberg School of Medicine, Chicago, IL, USA; ^8^ Chambers‐Grundy Center for Transformative Neuroscience, Kirk Kerkorian School of Medicine, University of Nevada, Las Vegas, NV, USA; ^9^ Chambers‐Grundy Center for Transformative Neuroscience, Department of Brain Health, School of Integrated Health Sciences, University of Nevada Las Vegas, Las Vegas, NV, USA; ^10^ Kirk Kerkorian School of Medicine, University of Nevada Las Vegas, Las Vegas, NV, USA; ^11^ Cleveland Clinic Lou Ruvo Center for Brain Health, Las Vegas, NV, USA; ^12^ Chambers‐Grundy Center for Transformative Neuroscience, Las Vegas, NV, USA; ^13^ University of Nevada, Las Vegas, NV, USA

## Abstract

**Background:**

Recent advances in single‐cell/nuclei multiome technology have enabled simultaneous profiling of gene expression and chromatin accessibility from the same nuclei, providing opportunities to interrogate the regulatory underpinnings responsible for Alzheimer's disease (AD) relevant transcriptomic and epigenomic features in a cell type‐specific manner.

**Method:**

This sequencing platform has successfully identified regulatory mechanisms responsible for AD‐associated transcriptomic changes in human cortical tissues. The cerebellum has traditionally received the most attention for its role in motor coordination. In this study, we conducted single‐nucleus multiome (snRNA‐seq and snATAC‐seq) profiles for postmortem human cerebellum and frontal cortex tissues with a varying neuropathologic degree of AD/ADRD. We used colocalization and fine‐mapping to identify likely causal target genes implicated in AD/ADRD cerebellum.

**Result:**

We report snRNA‐seq and snATAC‐seq analysis of 103,861 nuclei isolated from cerebellum from human cases of AD/ADRD and cognitive healthy controls, and with frontal cortex of AD donors for additional comparison. We identified disease‐associated granule cell subpopulations associated with AD/ADRD neuropathologies. Using peak‐to‐gene linkage analysis, we identified 431,834 significant linkages between gene expression and cell subtype‐specific chromatin accessibility regions enriched for candidate *cis*‐regulatory elements (cCREs). These cCREs were associated with AD/ADRD‐specific transcriptomic changes and disease‐related gene regulatory networks, especially for *RAR Related Orphan Receptor A* (*RORA*) and *E74 Like ETS Transcription Factor 1* (*ELF1*) in cerebellar Purkinje cells and granule cells, respectively. We further constructed transcription factor (TF)‐mediated gene regulatory networks in human AD/ADRD cerebellum and applied integrated trajectory analysis to characterize cerebellar granule cell states at the epigenomic and transcriptomic levels. Trajectory analysis of granule cell populations identified disease‐relevant transcription factors, such as *RORA*, and their regulatory targets. We identified two likely causal genes, including *SEZ6L2* in Purkinje cells and *KANSL1* in granule cells, through integrative analysis of cCREs derived from snATAC‐seq, genome‐wide AD/ADRD loci, and Hi‐C looping data from human cerebellum. CRISPR experiments show functional roles of *KANSL1 and SEZ6L2 in human iPSC‐derived neurons*.

**Conclusion:**

This comprehensive cell subtype‐specific regulatory landscape in the human cerebellum identified here offer novel genomic and epigenomic insights into the neuropathology and pathobiology of AD/ADRD and other neurological disorders if broadly applied.